# Beyond race: Recruitment of diverse participants in clinical genomics research for rare disease

**DOI:** 10.3389/fgene.2022.949422

**Published:** 2022-08-22

**Authors:** Jennifer L. Young, Meghan C. Halley, Beatriz Anguiano, Liliana Fernandez, Jonathan A. Bernstein, Matthew T. Wheeler, Holly K. Tabor, Undiagnosed Diseases Network Consortium

**Affiliations:** ^1^ Stanford Center for Biomedical Ethics, Stanford University School of Medicine, Stanford, CA, United States; ^2^ Center for Genetic Medicine, Northwestern Feinberg School of Medicine, Chicago, IL, United States; ^3^ Human Genetics and Genetic Counseling, Stanford University School of Medicine, Stanford, CA, United States; ^4^ Stanford Center for Undiagnosed Diseases, Stanford University, Stanford, CA, United States; ^5^ Department of Pediatrics, Stanford University School of Medicine, Stanford, CA, United States; ^6^ Division of Cardiovascular Medicine, Stanford University School of Medicine, Stanford, CA, United States; ^7^ Department of Medicine, Stanford University, Stanford, CA, United States

**Keywords:** rare disease, equity, genome sequencing, exome sequencing, pediatrics

## Abstract

**Purpose:** Despite recent attention to increasing diversity in clinical genomics research, researchers still struggle to recruit participants from varied sociodemographic backgrounds. We examined the experiences of parents from diverse backgrounds with enrolling their children in clinical genomics research on rare diseases. We explored the barriers and facilitators parents encountered and possible impacts of sociodemographic factors on their access to research.

**Methods:** We utilized semi-structured interviews with parents of children participating in the Undiagnosed Diseases Network. Interview data were analyzed using comparative content analysis.

**Results:** We interviewed 13 Hispanic, 11 non-Hispanic White, four Asian, and two biracial parents. Participants discussed different pathways to clinical genomics research for rare disease as well as how sociodemographic factors shaped families’ access. Themes focused on variation in: 1) reliance on providers to access research; 2) cultural norms around health communication; 3) the role of social capital in streamlining access; and 4) the importance of language-concordant research engagement.

**Conclusion:** Our findings suggest that variables beyond race/ethnicity may influence access in clinical genomics research. Future efforts to diversify research participation should consider utilizing varied recruitment strategies to reach participants with diverse sociodemographic characteristics.

## Introduction

Access to clinical genomics research for people of diverse sociodemographic identities is essential for achieving equity in the distribution of benefits from the knowledge gained. Unequal access to opportunities to participate in research is not in adherence with the principle of justice, which would require that all members of society benefit equitably from scientific advancement (Green et al., 2020) Furthermore, to the extent that diverse genetic ancestries correlate with sociodemographic categories of race and ethnicity, the lack of diverse participants in genomics research reduces the broad applicability of findings and limits classification of rare variants in individuals from underrepresented groups (Bonkowsky et al., 2018; Landry and Rehm, 2018)

Calls have been made to increase recruitment of racially/ethnically diverse participants in clinical genomics research since the vast majority of participants have been of European descent (Roberts et al., 2018; Ceyhan-Birsoy et al., 2019; Fatumo et al., 2022) The issue of equity in access has been particularly pointed in the context of rare diseases, for which patients face additional challenges related to care coordination and access to knowledgeable specialists (Splinter et al., 2018; Walley et al., 2018) Over the last decade, research has advanced the use of genomic sequencing for gene discovery and diagnosis of rare diseases, with the potential to improve access to genetic diagnosis for rare disease patients (Spillmann et al., 2017; Posey et al., 2019) Indeed, as many patients face insurance and other barriers to accessing genomic sequencing in clinical care, research has become a source of access to diagnostic tools such as sequencing for patients (Delikurt et al., 2015) As a result, in the context of rare disease, failure to reduce inequities in access to research also may contribute to health disparities in access to genetic diagnosis for rare disease patients.

Prior research provides some insights into barriers to research participation that disproportionately impact certain communities. A number of sociodemographic factors are known to shape access to health research, including cultural norms and beliefs related to health and illness, lack of education, financial resources and health insurance (Lee et al., 2019; McGuire et al., 2020; Fatumo et al., 2022) Especially for historically excluded or exploited groups, a lack of trust in research–and in healthcare institutions more broadly–has been reported as a central reason for declining to participate in research. There is evidence that this is particularly problematic in clinical genomics research, where certain racial/ethnic groups report concerns about privacy and whether genomic information will be used against them by the government, healthcare system, or law enforcement–issues that disproportionately affect certain groups (Amendola et al., 2018; Claw et al., 2018; Passmore et al., 2019)

Though many barriers to clinical genomics research have been identified, the experiences of diverse participants who have successfully enrolled in clinical genomics research remain less well understood. These individuals’ perspectives are valuable insomuch as they may speak to not only potential barriers, but also the facilitators to research access. Further, the literature on barriers to access in clinical genomics research has relied heavily on the lens of race/ethnicity, with less emphasis placed on the ways in which other sociodemographic factors may shape access.

To address these gaps, we conducted interviews with parents of diverse racial/ethnic backgrounds to examine their experiences of enrolling their children in clinical genomics research, including barriers and facilitators they encountered, and how various sociodemographic factors shaped their access to research. We focused on access to clinical genomics research for diverse patients with rare genetic diseases, including barriers and facilitators to identification of research opportunities, recruitment for and enrollment in studies, and participant retention.

## Materials and methods

### Study setting

We conducted this study in collaboration with the Stanford University clinical site of the Undiagnosed Diseases Network (UDN) (Reuter et al., 2018) The UDN is a research consortium developed to advance the science of rare disease discovery and diagnosis through a case-based approach (Gahl et al., 2016) Any individual may apply to the UDN, though a referring provider letter is required. Enrollment is based on multiple criteria, including the presence of an undiagnosed condition despite thorough evaluation by a health care provider, the presence of at least one objective finding, and willingness to consent to, travel for (when necessary), and participate in the recommended clinical, research, and genetic evaluation (Ramoni et al., 2017)

As of December 2021, the UDN had evaluated over 1,500 participants and diagnosed 505 of those individuals (Network, 2021) Applicants to the UDN are (>80%) White and Non-Hispanic. The network reports no difference in rate of acceptance for different racial/ethnic groups among those who complete the application (Walley et al., 2018) At the Stanford University site 49.5% of enrolled pediatric participants identified as Non-Hispanic White, 26.3% as White Hispanic, 11.8% as Asian, 8.2% as Multiracial, and 1.5% as Black or African American.

The UDN study is approved by a central institutional review board at the National Institutes of Health and is registered at clinicaltrials.gov (NCT02450851) (Splinter et al., 2018) This study was also approved by the Stanford University Institutional Review Board.

### Participant recruitment

A UDN clinical site coordinator provided the lead researcher on the study team with contact information for parents of UDN participants who previously agreed to be contacted for future research. We utilized quota sampling (Bernard, 2006) to ensure racial/ethnic diversity. We focused recruitment on Asian American and Hispanic families, the two largest racial/ethnic minority groups at the study site. We intentionally recruited non-Hispanic White participants for one-third of our sample as a comparison group. A Spanish-English bilingual clinical site coordinator and bilingual researchers helped to recruit Spanish-speaking parents. Researchers contacted potential participants through phone and email. Individuals were eligible to participate if they were the parent or legal guardian of a current UDN participant and if their primary language was either English or Spanish.

### Data collection

Parents who consented to participate in the study completed a single in-depth, semi-structured interview in either English or Spanish, lasting between one and 2 hours. After reviewing the literature, the study team developed the interview guide and pilot tested it with parents with children with undiagnosed or rare diseases. The final interview guide included questions regarding the participant’s sociodemographic characteristics, family structure, their child’s diagnostic odyssey, and experiences before, during and after participating in the UDN, including barriers to and facilitators of access to research. Interviews were audio-recorded and transcribed verbatim, translated from Spanish to English (when necessary), and de-identified for analysis.

### Data analysis

The research team analyzed interview data using Dedoose software (Dedoose (9.0.17), 2021) Three experienced qualitative researchers (JLY, MCH, HKT) utilized inductive and deductive approaches to conduct a comparative content analysis of the data (Miles et al., 2018) First the research team reviewed the transcripts to define deductive codes related to broad content area (e.g., “family,” “healthcare experiences,” “genetic testing experiences”). We conducted repeated interrater reliability testing in the application of these codes until the average pooled Cohen’s kappa reached *κ* > 0.8, indicating excellent agreement (Miles et al., 2018) We then applied the deductive broad codes to all transcripts. Drawing on inductive techniques from Grounded Theory (Strauss et al., 1998), we then used these codes to iteratively explore potential mediating factors driving similarities and differences in participants’ experiences accessing research by various sociodemographic characteristics, including race/ethnicity, primary language, education, and income. This process included attention to both expressed (emic) differences in access as well as observed (etic) differences within and across different subsets of our sample (Strauss et al., 1998)

## Results

### Participant characteristics

We completed interviews with one parent from 30 unique families. Twenty participants completed interviews in English and 10 participants completed interviews in Spanish. Parents self-identified as Asian (*n* = 4), non-Hispanic White (*n* = 11), Hispanic (*n* = 14), and multiracial (n = 2). Participating parents were predominantly female (n = 27, 93.3%), and diverse in terms of income and education. See Table 1 for full sample characteristics.

**TABLE 1:** Sample characteristics.

	N		%
**Parent gender**			
Female	27		93.3
Male	3		10.0
**Parent Race/Ethnicity**			
Hispanic (any race)	13		43.3
White (Non-Hispanic)	11		36.6
Asian-American (Non-Hispanic)	4		13.3
More than one race/ethnicity	2		6.6
**Parent Preferred Language**			
English	20		66.6
Spanish	10		33.3
**Household Combined Income**			
< $50,000	7		23.3
$50,000 - $100,000	7		23.3
$100,001 - $150,000	9		30.0
$150,001 - $200,000	4		13.3
> $200,000	3		10.0
**Highest Education Completed**			
No school	1		3.3
Elementary school	1		3.3
Some high school	5		16.7
High school	4		13.3
Some college	3		10.0
College	7		23.3
Graduate degree	9		30.0
**Number of Children in UDN**			
One	23		76.7
Two	6		20.0
Three	1		3.3
**Child(ren)’s Diagnostic Status**			
Diagnosed	13		43.3
Undiagnosed	14		46.7
Emerging/Candidate Diagnosis	3		10.0
**Total All Parent Participants**	**30**		**100**

Bold values are the our participants were all parents, this is how we describe them in the table in comparison to any data about their children.

### Barriers and facilitators to clinical genomic research in rare disease

The results of our analysis are organized into four themes regarding aspects of participants’ experiences with clinical genomics research that suggest potential barriers and/or facilitators to access. While the first two themes relate to participant race/ethnicity, the second two highlight the extent to which additional sociodemographic characteristics, beyond race/ethnicity, also may shape families’ access to clinical genomics research. Specifically, themes highlight variation in: 1) reliance on providers to access research; 2) cultural norms around health communication; 3) the role of social capital in streamlining access; and 4) the importance of language-concordant research engagement. Below we describe these four themes, the connections across themes, and provide supporting quotes.

### Theme 1: Reliance on providers to access research

The first theme focuses on the role of healthcare providers in facilitating access to research. Hispanic parent participants more commonly reported relying on and trusting providers to help their child and to facilitate access to clinical genomic research. This trust existed across income and education levels, as well as English language proficiency. These parents shared a distinct description of gratitude towards providers for their persistence in searching for a diagnosis, finding therapies, and helping families navigate the medical system. This was especially relevant for parents who reported struggling to understand complex medical information and did not feel confident in their ability to provide their child with appropriate care. For example:

*There’s a lot of need and more necessity in our culture because there’s a [lack of knowledge]. We don’t understand that there are different diseases that we don’t know about. So it is up to the professionals not to give up. If God gave them that knowledge to research, to study, it is so they can help more people live a life that perhaps is not normal, but is better. (P29, Mother, Hispanic)*

In responding to a question about what advice they would offer to other families trying to access research, participants also described the importance of a close relationship with providers.

*What I would say is … believe in the doctors. Believing that there are people who are interested in helping others, in this case the doctors who are interested in our children (P20, Mother, Hispanic)*

One parent described how this relationship could be especially important for families with language barriers.

*Especially for the families that don’t speak English, I would say really to have a close relationship with your primary provider–primary care. And that you feel that you could tell them anything, I think that is key. I feel a lot of parents they know something’s wrong but they don’t know how to take care of it. When my daughter was sick I didn’t know, I was young, I was naïve, I didn’t know. (P04, Mother, Hispanic)*

The degree of trust in providers prominent in the narrative of Hispanic participants was distinct from that of White and Asian parents. Non-Hispanic families more commonly described compromised trust in providers, and/or the healthcare system as a whole.

*We didn’t trust doctors for a very long time. (P02, Mother, Asian)*

*That was definitely an eye opener because I trusted our provider so much … so much. And I think it broke a lot of trust for both my husband and myself. (P15, Mother, White)*

As discussed further below, White and Asian parents in our study population were more likely to report higher incomes and education levels, and this access to social capital appears to have facilitated more direct access to clinical genomics research despite distrust.

### Theme 2: Cultural norms around health communication

The second theme focuses on variation in reported cultural norms around health communication. More frequently in Asian participants, communication–or lack of communication–about a child’s illness was described as shaping the ways in which they accessed research opportunities. Specifically, three out of the four Asian participants independently described a tendency for individuals in the Asian community to conceal or avoid discussing issues related to illness or disability, even among close family members. For example, P01 shared that, “it would be definitely harder for [an Asian family] to speak and talk about the situation,” and described how she herself has struggled to communicate with her parents about her child’s condition. She also shared a story of a friend and fellow Asian mother who also had only told a few people about her child’s rare disease.

Though participants narratives focused on health communication within families, they also identified implications of these cultural norms for access to research and suggested strategies to overcome this barrier. For example, P02 pointed out that putting information online is important to families who are less likely to discuss their child’s condition with others.

*Get an [Asian] family to talk about a medical issue, it is not going to happen. They will hide, hide, hide. They will not be as open with sharing the data, but they are research oriented. Best thing is to put it online, they will Google the condition. Word-of-mouth will not work with 60–70% of Asians. (P02, Mother, Asian)*

This challenge was not reported among other participants, who described more open communication among immediate and extended family as well as other support networks. In contrast to P01, a White mother (P16), said that “we don’t keep [our child’s] care or diagnosis or, you know, journey or anything a secret from anyone.”

### Theme 3: Role of social capital in streamlining access

The third theme focused on the role of social capital–including existing social networks and the ability to find and navigate such connections–in facilitating some parents’ ability to directly access clinical genomics research. This theme was prominent in English-speaking participants of varying racial/ethnic backgrounds with high levels of education and family income, and notably absent among participants with lower levels of education and income.

For example, one parent who worked in the sciences independently identified the opportunity to participate in clinical genomics research by talking to physicians at her work about her child.

*When I talked to physicians at my work, and there’s one in particular … I told her who I had reached out to and she’s like that’s perfect, go to the UDN. (P07, mother, White)*

Another parent identified the opportunity to participate in research through a non-profit organization to which her family had given charitable donations in the past.

*It was my husband who actually connected with [non-profit organization] and then started donating to that network–I think that’s how we got connected with the UDN and then got enrolled. (P13, mother, Asian)*

Another parent found out about the opportunity to participate in clinical genomics research while he was attending a fundraising event, during which he made the acquaintance of someone directly involved in clinical genomics research.

*I basically met [a doctor’s wife] at an event. At the conclusion of the event I just met her serendipitously and we were talking about kids, and I mentioned S (my daughter), and we talked a little and she said, well, you got to meet [my husband] because [he] found out last week about an $80 million National Institute of Health funded study for people with undiagnosed medical conditions. (P19, father, White)*

Other parents focused on their own efforts and abilities in independently gathering information and navigating potential research opportunities. These individuals all reported having a master’s degree or higher, demonstrated a high level of genetic literacy, and described doing extensive research.

*And then [the providers] went to [genetic testing] panels … but now we’re going to do whole exome, and then finally, like we were not offered whole genome but that’s when I was like, well, we’ve had two rounds of this, like I want to go somewhere else. And so that’s when we went to the UDN. (P14, Father, White)*

*I was kind of doing my own research and I was like, wait, I think I’ve heard of this before. And I went back and (our geneticist) told me about this! And so then I called from there. I really focused on it for the first few years, like just pursuing and pursuing, and since that had been my focus we had a lot of data already which helped I think get us in. (P06, Mother, Biracial)*

Not all parents in the highest income brackets and highest level of education groups discussed leveraging social capital to directly access research. However, no families without such resources described doing so and some even noted how difficult this might be.

*I see [other] parents and they look for resources, it is like, I just don’t have the bandwidth. I’m just so exhausted. (P05, Mother, Hispanic)*

### Theme 4: Importance of language-concordant research engagement

The fourth theme focuses on the importance of language-concordant communication in facilitating recruitment from diverse patients who had limited English proficiency (LEP). These parents stressed the value of Spanish language communication in accessing research. One LEP parent described how having a Spanish-speaking provider serve as a navigator during the application process for the UDN helped access this resource:

*The geneticist, she’s a very good person, told me: “Look, here’s the form, fill it out.” At that time there was a speech therapist with my daughter and she helped me to fill the form out and entered her over the computer. (P27, Mother, Hispanic)*

Another parent highlighted the importance of Spanish language services particularly in the context of genetics, due to the complexity of the conversations required. She said that although she does not always require interpretation services:

*…when I go to genetics, I ask if they can give me an interpreter because the doctor talks using numbers and codes that I don’t understand. But normally I do the appointments alone. (P24, Spanish-speaking mother)*

Though this participant is referring to the clinical context, her comment also has implications for clinical genomics research.

Retention of participants is an equally important aspect of access to research and Spanish-speaking parents spoke very positively about the communication and support they received from a Spanish-speaking research coordinator working for the Stanford UDN.

*We’re happy with (Spanish-speaking UDN staff) who has been talking with us, she’s telling us every time they find something or if they look at something or if they have not found anything yet, but they keep letting us know. They make sure we don’t think they forget her. (P25, Spanish-speaking mother)*

This experience among LEP parents stood in contrast to those described by many of the English-speaking parents. Indeed, for English-speaking parents, a lack of consistent and clear communication was their primary complaint regarding their experiences with clinical genomics research.

*I would love for [the UDN] to communicate more often … I don’t want to feel like a second afterthought. (P08, Mother, White)*

While we cannot conclude that Spanish language was the only driver of increased satisfaction, given the differences by language across our sample, this suggests at least a reasonable hypothesis. Providing language-concordant support in navigating study participation appeared to improve the quality of communication between participants and researchers beyond even what was experienced by English-speaking participants.

## Discussion

Our results describe potential barriers and facilitators to accessing and participating in clinical genomics research for parents of children with rare diseases from sociodemographically diverse backgrounds, including relationships with providers, cultural norms around communication, and access to social capital and language-concordant research resources. Parents’ narratives highlighted sociodemographic factors–including income, education, cultural norms, and language proficiency–that may play a role in shaping access, either separately or in addition to race and ethnicity.

In this study, Hispanic participants more commonly stressed the importance of having a close and trusting relationship with a provider in facilitating access to research opportunities. In contrast, Asian participants more frequently described cultural barriers to communication but also identified online resources as a potential avenue identifying research opportunities. However, it was only the subset of participants who had high income and education that reported being able to leverage social capital to facilitate access to research. The role of cultural norms, language, education, and income illustrated in our themes highlight the importance of examining multiple sociodemographic characteristics in addition to race/ethnicity when considering barriers and facilitators to clinical genomics research.

Our results align with literature on the role of communication in clinical research, and the extent to which the quality and consistency of communication may influence patient identification and enrollment practices (Sae-Hau et al., 2021) Language-concordant engagement has been shown to facilitate navigation of clinical genetic services for individuals with limited English proficiency (Pacyna et al., 2021; de Leon et al., 2022) Our study suggests that resources for communication may be equally important in genomics research, where concepts and terminology are very complex and non-native English speakers who do not typically need an interpreter may struggle with communication. Given that nearly one in 10 adults in the United States have limited English proficiency, language-concordant research services are an essential tool for inclusive research (Ryan, 2013)

Our findings also intersect in unexpected ways with the literature on the role of trust in recruitment of historically marginalized communities in clinical genomics research (George et al., 2013; Claw et al., 2018; Kraft et al., 2018; Lee et al., 2019; Armstrong and Ritchie, 2022) While this literature has emphasized trust–and specifically mistrust–as a barrier to clinical genomics research participation, Hispanic parents in our study expressed *more* trust in providers to identify and refer them to clinical genomics research than other parents. Prior research suggests that those who are less familiar with the bureaucracy of clinical genomics research may rely more heavily on providers and researchers to facilitate access (Levine et al., 2001) Research also points to the important role of providers in helping patients access clinical genetics services and could be expanded to understand how these providers may also facilitate participation in clinical genomics research (Chou et al., 2021) More research is needed to understand the barriers that these providers themselves may face in equitably referring patients for clinical genomic research.

### Implications for clinical genomics research

Recently, leaders in the scientific community have pledged to address structural racism in biomedical research through efforts focused on diversifying the genetics workforce and research participant populations (Kaiser, 2021), changes to publication policies (Brothers et al., 2021), and to research funding (Health NIo, 2021) In addition to these efforts, our findings point to the need for an intersectional approach to recruitment and retention. Rather than using race/ethnicity as a proxy for other sociodemographic characteristics, clinical genomics researchers should plan to systematically collect a broader range of variables associated with structural inequities, such as income, education, and language proficiency. Tracking this data throughout study design, implementation, and analysis is critical to promoting greater inclusion of participants from underrepresented populations that face compounding barriers to research (Bentley et al., 2017)

Recruitment strategies must address heterogeneity in access to research. Participants in our sample varied in their pathways to accessing clinical genomics research. Some relied heavily on their clinical providers to identify opportunities and facilitate access, while others leveraged social connections and/or their own research to identify these opportunities independently. Investigators developing recruitment strategies could leverage these varied approaches to reach diverse patient populations. Online resources such as clinicaltrials.gov may be a key resource for some families but less accessible to those with limited education or English-language proficiency. For other families, tools such as patient navigation may be more helpful (Fouad et al., 2016; Uveges et al., 2018) In clinical trials research, patient navigators have been utilized to meet individual needs and address barriers or concerns of participants enrolled (Ghebre et al., 2014; Uveges et al., 2018) These programs also have the potential to serve as a conduit between clinical providers and researchers. However, research is needed to effectively translate these models into clinical genomics research for specific populations.

Clinical genomic researchers must exercise caution to avoid privileging access to research participation to only those who have the resources and skills to independently identify research opportunities. Funding agencies, such as the National Institutes of Health, could promote or incentivize the adoption of research recruitment and retention efforts that are compatible with the language and cultural diversity present in the populations.

### Limitations

While the racial/ethnic diversity of our sample is a strength, our study did not include the important perspectives of other groups such as Black patients and families due to small numbers at the study site as well as lack of response to recruitment efforts. The Asian American sample was also small and included people who identified as East Asian and South Asian, thus our conclusions about this subgroup may not be broadly generalizable to the many heterogeneous cultures represented by the term “Asian.” In addition, our participants include those who were able to successfully access clinical genomics research. Although we explicitly probed for insights into reaching diverse communities more broadly, directly soliciting the experiences of those who were not able to access research, or who chose not to participate, remains critical. This is a small sample from a single clinical genomics study and further research with larger samples is needed to examine how diverse patient characteristics relate to research access, recruitment, and retention outside of the context of rare disease and clinical genomics research for diagnostic purposes.

## Conclusion

This study suggests multiple sociodeomographic factors–including and in addition to race/ethnicity–may related to barriers and facilitators to clinical genomics research. Future research must look beyond race/ethnicity variables to better incorporate factors such as education, income, social networks, and cultural norms in planning recruitment and retention efforts to expand accessibility. Research on the ethical, legal, and social implications of genetic and genomic research often recruits convenience samples from larger genomics studies and must avoid the pitfalls and biases of convenience sampling. Facilitating research participation for all people, not just those who have sociodemographic advantages, will ensure equitable access to the direct benefits of research such as receiving a potential genetic diagnosis, as well as the indirect downstream benefits of generalizable knowledge in genomic research to communities and patients that have historically been excluded.

## Data Availability

The raw data supporting the conclusion of this article will be made available by the authors, without undue reservation.
